# Anomalous Case of Enteritis, with Remarks

**Published:** 1824-08

**Authors:** Edward Blackmore


### Art. III.—*Anomalous Case of Enteritis, with Remarks.*

By Edward Blackmore, M.D.

November 8, 1822.—H. Williams, æt. thirty-eight, a labourer, strong, muscular, has been sickening, with slight pain at the stomach, for several weeks, during the last of which the pain has been severe: it is not increased by taking a full breath, and seems to be relieved by pressure; belly not tense or tumid; pulse 80; vomiting; constipation; urine high coloured. Bled to twelve ounces; enema purgans; jalap *c* cal.

9th.—A scanty stool from two glysters, pain aggravated after the second; thirst and fever. Injection of folior. nicotian. tabaci ʒj. infused in a pint of water. Injection caused faintness, sweats, and two mucous stools; pain at heart continues, with retchings.

10th.—No dejection, no pain on pressure; pulse 64, tongue fouler. Ol. ricini, ℥j. *c* Tr. opii, ʒss.

Evening.—No dejection, no pain; feels exhausted. Cathartic continued.

11th.—Three stools; slight rigor, and gnawing pain at navel. Cathartic continued.

12th.—More pain and tenderness; vomiting; belly tumid; great tenesmus. Bled to ten ounces.

13th.—Urgent vomiting with severe pain, which is relieved by hot fomentations; tenesmus; no stool since the 11th. Mustard cataplasm to belly; anodyne injection.

Mid-day.—Seen in consultation with my friend, Dr. Cookworthy. Much tenderness of belly; pulse 64, full and firm; tongue furred and white; some stupor from the laudanum injection. Bled to twenty-five ounces. Pulse rose to 98, softer. Calomel, gr. j. every four hours. No hernia exists.

Evening.—Pulse 64, tense; no vomiting since the morning; sickishness and eructation. The pain has changed its seat, and is now affected by pressure. Voice weak and plaintive; urine scanty, red, not turbid; no stool for forty-eight hours; belly not tumid, but there is slight hardness at the navel. Bled to twenty ounces, when the pulse rose to 100.

14th.—Much pain through the night, relieved by a jar of hot water to the belly; pain is deep-seated, and increased by pressure and on taking a full breath; no stool; great tenesmus; urine scanty and red, with much sediment, like cretaceous powder; tongue furred, white; belly not tumid; slight retching; pulse 96, tense, and not small. Bled to twenty-four ounces, when he became deadly faint and convulsed; pulse at wrist gone for three minutes; revived by aspersion of cold water. In ten minutes, pulse 120, small; pain quite gone for some time, then again felt. Continue calomel, gr. j. every four hours; salts and senna.

Evening.—Pulse 116, tense and small; belly more tense and tumid; strong retching, hiccup; pain still increased by pressure. A tobacco injection caused giddiness, but did not affect the pulse. No stool for seventy-two hours; urine red, with copious sediment. Has taken calomel gr. xj.; vomited; senna. Twelve leeches to belly, and cataplasm afterwards.

15th.—Slight pain on pressure; belly still tumid, with hardness on the right side; pulse 112, less sharp; vomiting of a clear fluid; a whitish, scruppy, fecal dejection, from the tobacco injection; look and voice improved; tongue furred, brownish. Twelve leeches; glyster of salts and senna. Calomel, gr. j.; Antim. pulv. gr. iij. every four hours.

Evening.—Much tenderness at lower part of the belly; slight retching; pulse 120, compressible; urine copious, less red and turbid. Three stools of small fecal particles. Glysters and medicines repeated.

16th.—Many feculent stools, much turbid urine; slight ptyalism and mercurial fetor; some tenderness of belly; tongue brown; thirst and faintishness; pulse 112, small and soft; has taken calomel ℈j. Continue calomel and antim.; mist. salin. ex citrat. potass.

Evening.—Pulse 98, soft; belly bears strong pressure; slight colic; many dark feculent stools; much urine, of lighter colour. Extract. humali lupuli, gr. x.; ol. ricini, ℥j.

17th.—Pulse 100, soft; many dark bilious stools; tongue fouler. Omit calomel and antim. Continue ol. ricini, &c.

18th.—Many stools; tongue fouler. Continue mist. salin.

20th.—Two large formed stools; slight pain at heart, from eating an apple-dumpling; pulse 104, full. Mist. purgunt. ℥iij.

21st.—Mouth sore; convalescent.

None of the blood drawn was at all buffy or sizy; the crassamentum was convex, and not very firm. The blood coagulated at once, and was all of the same colour.

### *Reflections on this Case.*

1. In attempting to refer this disease to its proper seat in Cullen's Nosology, I find that one very important character of his class, Phlegmasiæ, is wanting,—" sanguis missus superficiem coriaceum album ostendens." When the case was first seen, it had all the characters of Cullen's definition of ileus or colica, and I should refer it to species 3, Colica Stercorea, or Ileus, a fecibus induratis. Some medical writers err in supposing that stercoraceous vomiting is essential to constitute this disease. Celsus simply remarks, " Si superior pars intestinorum affecta est cibus, si inferior pars sterca, per os redditur." And Van Swieten terms constipation of the bowels from inflammation, ileus. In one point, however, the character of colic was wanting in the present instance: the pain did not, from a state of ease, become acute. (*Pemberton*, page 175.) Although there was not a strong expression of pain, I believe there was constantly an obtuse pain at the navel. Is the disease then to be considered inflammatory? It wants the character, "dolor abdominis pungens," of Cullen's general definition of enteritis; nor has it the character, "pyrexia vehementi," of the species phlegmodea. It has the character, "pyresia lexion," of the species erythematica; but it wants that "cum diarrhœa!" Moreover, the pain is not tensive, that of the phlegmasia; nor burning, that of the erythema, which is the sort of inflammation that for the most part affects mucous membranes. The disease also wants Good's character of enteritis, "tension and tenderness." The pulse wants Baillie's character, "frequent, small, hard:" it is slow, large, and hard, more like the pulse of acute rheumatism.

The little increase of pain on pressure, and the want of tension, are given by Pemberton as characters of disease of the mucous membrane of the intestines: these are seen in this case, but other important marks are absent. The disorder is not peritonitis, for in this the tenderness and tension of the belly are distinctly marked.

The nature of the disease might seem at first to be obscure; the symptoms are perplexing; many characters usually considered to mark the existence of inflammation, are absent. The inflammatory character of the disease is, indeed, more manifestly developed on the 13th; but I am inclined to think that inflammation existed from the first day I saw the patient, and that its seat was the muscular coat of the intestines.

First. We have satisfactory evidence that inflammation may exist in the intestines to a fatal extent, where there has been little, if any, expression of pain. This is a most important fact, and it should be strongly impressed on the minds of young medical practitioners especially, since the statements of some of the most popular medical authors are calculated to mislead in this particular. Thus, Dr. W. Philip remarks, in his "Symptomatic Fever," page 225, "Our best diagnostic of pain attending enteritis is, that it is greatly increased on pressure." Now let us hear Morgagni, Ep. xxxv. art. 20, "Nec tamen si quando alterum vel utrumque horum (dolorem et acutum febrem), aut abesse aut vie esse invenies, continui putabis; aut nullum esse inflammationem aut levem; neque gangrenam et sphacelum in eorum esse intestini, non posse, in quibus duo illa præcecisse non videbis. Albertinum mihi inculeaverit, post leves dolores, nulla manifesta febre, nullo vomitu, animo ac corpore satis vigentibus, de inopinato vidisse ægros in præceps ruere et citò eripi ab latenti inflammatione. Se periculum intelligere ex pulsu humili et debili, sibi sub obscure dissimili; ex abdomine tenso, duro, cum dolore quodam; ex facie insoliti aliquid, sed in aliis aliud, ostendenti!"

Second. The existence and the degree of inflammation of many vital organs, cannot be judged of by the degree of existing pyrexia.

Mr. Hunter has beautifully remarked on the great diversity of the constitutional disorder arising from inflammation situated in vital organs, and in those not vital. In the one case, the whole system is in an uproar,—all is energy and activity; in the other, the system is calm, and submissive, and depressed, as if conscious it was assailed by a foe, whom to resist would be only to exasperate, and render the fatal issue of the conflict more sure and speedy.

Again, it is established, by the observations of the best pathologists, that the state of the pulse, as to frequency, cannot be relied on as affording an evidence of the presence or absence of local inflammation. Baillie, speaking of strangulation of the intestine, remarks, (*Morb. Anat.* p. 212,) "The pulse sometimes is not increased in frequency beyond the standard of health, and yet the inflammation of the bowel has been discovered, by the operation, to be very great." Prosper Alpinus admonishes, "Id memoriæ mandandum, ut nunquam ex solis pulsibus malum judicium feramus; sed aliis signis unà conspectis ac simul collatis." Abercrombie also makes these practical conclusions, from very sufficient premises, "Extensive inflammation may be going on, with every variety in the pulse, and without constant pain." Fordyce accounted the "hard pulse a pathognomic symptom of coma, or inflammatory fever, and that this state is characterised by buffy blood; whereas, in the obstructed pulse, the blood coagulates at once, and is all of the same colour." In the present case, we have the firm pulse without the buffy blood. Nor is the remark of Sinnertus exactly fulfilled here, "Pulsus validus et vehemens facultatem fortem monstrat; simul irritationem magnum significat:" at least, this case shows that the converse of this proposition is not invariably true, and that there may exist much vital power, (as is manifest from the large abstraction of blood requisite to impress the system,) and great irritation, without the pulse being strong and vehement.

Lastly. A buffy state of blood is not invariably connected with the existence of inflammation. Van Swieten remarks, (*Comment.* vol. iii. p. 47,) "Adsunt ergo talis sanguinis diathesis, licet nulla inflammatio adesset, at contrà, in validissimis inflammatoriis morbis aliquando nulla talis crusta in sanguine apparint; quod tamen pro malo omine semper habita fuit."

It is of importance to remember that the constitutional disorder consequent on local inflammation, or irritation, greatly depends on the habit of body in which the inflammation occurs. In delicate, sensitive, irritable bodies,—such, for example, as those of children and females tenderly brought up,—we should expect, and we find, the general disorder to be great, even where the local disease is small: while in stout, muscular, hardy bred labouring men, it requires a very powerful irritation to rouse the torpid actions of the system. In looking for the concomitants of disease, and estimating the power of remedies to be employed, these considerations must never be forgotten.

II. On reviewing the treatment of the present case, it will be remarked that purgatives were very sparingly used, before the inflammation was thought to be subdued by large blood-lettings. This to some may need a little vindication. In the general treatment of diseases, it is usual to employ two or more various means which agree in fulfilling the same intention, and produce a like ultimate effect. Thus, purging is held to be a useful auxiliary to blood-letting, in subduing inordinate vascular action. But it should be considered whether, from the particular seat or accompaniments of the disease, that means which is ordinarily an auxiliary in the curative plan, may not be inexpedient in the particular example. For instance, vomiting is a useful auxiliary to blood-letting, in the cure of many inflammations; but, if there exist a disposition to coma from determination of blood to the head, an emetic is said to be counter-indicated. Let us consider whether purging be not, in like manner, counter-indicated when inflammation of the intestine exists. In this state there is obstinate constipation, ςενοχωρια, "cura via ob carnem annatam vel tumorem in meatu existentem clauditur!" And it seems to be a maxim of sound reason, "evacuationes per alvum nunquam excitandæ, nisi viæ ad materiam emittendam propriæ patescunt." (Home, *Principia Medicinæ*, p. 49.) Now, what is the state of the lumen of an inflamed intestine? "Probatur naturaliter satis angustum esse intestinorum cavum: ubi ergo inflammatorius tumor nascitur in quodam intestinorum loco, non mirùm integrè claudi illud spatium, ita ut nihil transmitti possit." (Van Swieten, vi. 261.) "Tali ergo obstaculo nato, quod transitum impedit, sistuntur ad hunc lomen omnia ingesta." And what is the action of a purgative on the intestines? "Pars intestini lacissita a flatû aeri aspero constringetur, etiam a morte, vehementissime, in sede cui stimulus adplicatur, liberat se distendenti infesto corpore, id expellit in partem proximum laxi intestini; quæ eadem vi stimulo iterum contracta, repellit utrinque acceptum. Hic motus peristalticus, modo in una moxo in alia intestini sede peragitur, absque certo ordine, ubicunque aer cibusve stimulum admoverit." (HALLER, *Primæ Linæ.*) Now, if a purgative thus excites the movements of the intestines, and derives more blood to them by its irritative effect, and brings their contents to a part which they exasperate, but cannot pass, must it not augment the inflammation and the inverted action of the intestines, and lead to stercoraceous vomiting? How oftentimes do we remark an increase of pain in such cases, on the operation of a purgative. "At si colicum affectum," remarks Trallian, "ob inflammationem intestini fieri contingat, neque omnino exhibere audebis purgationem in his, et præcipue in initio cum inflammatio nondum ad coctionem pervenerit. Qui enim medicamenta ventri ducendo apta præbere non dubitârunt, ægrotantibus periculorum autores et mortis extiterunt!"

For the contrary practice, it may be urged that, in many cases, the retained fæces are the cause of the inflammation, and at least must aggravate the inflamed part; and, therefore, as you should extract a thorn from the flesh, you ought likewise to evacuate the irritating fæces. In opposition to this, I refer to Case v. in Dr. Abercrombie's paper on Ileus, in the Edinburgh Medical and Surgical Journal, a case very similar to the present, where the intestine was found inflamed, distended, and gangrenous, but the diseased part contained no consistent fæces! And I see no reason to believe that there ever exists in the small intestines consistent fæces, which may be a mechanical irritant to them, in the way that sequelæ are in the dysenteric affection of the large intestines. I cannot think that purgatives are proper in any inflammatory affection of the intestines, except only in chronic dysentery; and then the mildest alone should be employed. I decidedly dissent from the precept of Home, in his Principia Medicinæ, "Purgantibus lenissimis uti, et, si hæc nihil profuerint, acrioribus!" The warm bath, enemata, and local blood-letting, would be most proper to be used in the alternative.

In similar affections to the present example, opiates are often combined with purgatives, to keep them on the stomach; and an effervescing draught is given to allay the vomiting. I doubt the propriety of both these measures. The vomiting is an effect of the disease, and, for the most part, a correct index of its violence and progress. It surely cannot be right to rob ourselves of such an evidence, and lull ourselves into repose in the confidence that the danger is over, because it ceases to be apparent. In simple cases of colic, indeed, without inflammation, it is right to settle the stomach, in order to keep medicines on it; as, when the bowels are opened, the disorder is generally at an end: and, to effect our purpose, nothing is more useful than to exhibit the medicines in the smallest bulk and the driest form, positively forbidding any liquid to be drunk. "Anti ex inferiori parte spiritus transmittitur magna cura vitandum est ne quid bibat." (CELSUS.)

I consider the precise seat of the inflammation in the present case to be the muscular coat of the small intestines. Bichat has taught physicians to use more exactness in assigning the seat of diseases; and we now believe that inflammation may exist in one texture, muscular, serous, or mucous, for some time before it spreads to the adjoining textures. Little advance has, however, been made in this very exact pathology. Dr. Abercrombie has remarked, that the symptoms and the morbid appearances of ileus will accompany enteritis only when the inflammation is seated in the muscular coat; and, on the case before referred to as similar to the present, he asks, "Was the inflammation confined to the muscular coat, producing ileus, but not accompanied by symptoms of acute inflammation, till a few hours before death?" We well know how inflammation of the muscular texture destroys its action; if the substance of the heart is inflamed, the pulse is confined and unequal, and at times, for a few minutes, ceases altogether. It appears also to be the character of inflammation of the muscular texture, that it is of slow progress; and that it exists to a slight extent for some time, without great constitutional disturbance. This is seen in some varieties of rheumatism.

The obscure character of the present case at the beginning misled me into a practice, if not hurtful, at least inefficient. It will teach me anxiously to scrutinize every example of disease that is not fully developed, and that has not all the usual accompaniments. It impresses on me the wisdom and value of the admonition of Sydenham: "If we so diligently penetrate into the secret recesses of every disease, as to be able to discover it lying hid under irregular symptoms, it will presently appear of what species it is, and be easily referred to the sort to which it belongs. Moreover, the method whereby such diseases are to be treated must be accommodated not to counterfeit symptoms, but to the disease itself, whatever it is, as if it were perfectly formed, and naturally existing." (PECHEY's *Sydenham*, p. 204.) It is well to understand our errors, and gather wisdom from our ignorance. He is not the wise physician who assigns a name to a disease, and exhibits remedies for its cure, because it has been so done before him. Nor is he the experienced physician, who has merely named and prescribed for any number of cases, ever so large. But he is wise and experienced who has truly observed the nature of maladies, and who knows to select the remedy suited to every various state of disease, and on whose mind the few important and rational principles of medicine have been deeply imprinted by important facts. "Ainsi le meilleur medicin n'est pas, comme le prejugé suppose, celui qui accumule en aveugle et en courant beaucoup de pratique, mais celui qui ne fait que des observations bien approfondies, et qui joint à ces observations le nombre beaucoup plus grand des observations faites dans tous les siècles par des hommes animés du même esprit que lui. Ces observations sont le veritable experience du medicin." (D'ALEMBERT.)

*Plymouth; May* 1824.

